# Effects of Cannabidiol‐Rich Cannabis Oil on Facial Expression Recognition and Theory of Mind in Children With Autism Spectrum Disorder: A Randomized Clinical Trial

**DOI:** 10.1002/aur.70353

**Published:** 2026-08-26

**Authors:** Wandersonia Moreira Brito Medeiros, Estácio Amaro da Silva Junior, Katy Lísias Gondim Dias de Albuquerque, Marine Diniz da Rosa, Amanda Gleiciane de Lima Oliveira, Maycon Jônatas da Silva, João Vitor Andrade Fernandes, Emily Caroline Costa Vieira, João Paulo Mendes dos Santos, Thais Cavalcanti Borges, Ana Hermínia Andrade e Silva, Carlos Espínola Neto Segundo, Nelson Torro‐Alves

**Affiliations:** ^1^ Federal University of Paraíba João Pessoa Paraíba Brazil; ^2^ University Center Maurício de Nassau João Pessoa Paraíba Brazil

**Keywords:** anxiety, autism spectrum disorder, cannabidiol, children, randomized controlled trial

## Abstract

To evaluate, in a post hoc subanalysis, the effects of cannabidiol (CBD)‐rich cannabis oil on facial expression recognition and theory of mind in children with autism spectrum disorder (ASD). This post hoc subanalysis included children from a double‐blind, randomized, placebo‐controlled trial who completed social cognition assessments at baseline and after treatment. Participants received CBD‐rich cannabis oil or placebo for 12 weeks. Facial expression recognition and theory of mind were assessed using neuropsychological tasks. Between‐group differences were evaluated using the Mann–Whitney U test, whereas within‐group changes were assessed using the Wilcoxon signed‐rank test. Nineteen Brazilian children with ASD were included (72.7% male; mean age, 7.82 years). Within‐group analyses showed significant improvements in facial expression recognition (*p* = 0.030) and verbal theory of mind (*p* = 0.016) after treatment in the CBD group, whereas corresponding changes in the placebo group were not statistically significant. However, between‐group comparisons showed no significant differences after treatment in facial expression recognition (*p* = 0.527) or verbal theory of mind (*p* = 0.901). Total theory of mind scores improved significantly within both groups, with no significant between‐group difference (*p* = 0.967). Although significant within‐group improvements in facial expression recognition and verbal theory of mind were observed following CBD‐rich cannabis oil treatment, these changes were not significantly different from those observed with placebo. Given the small sample size and exploratory post hoc design, these findings should be interpreted cautiously and do not establish a treatment effect of CBD on social cognition in children with ASD.

**Trial Registration:**

Brazilian Clinical Trials Registry (ReBEC), RBR‐5wr2cqq (UTN: U1111‐1261‐4178), accessible at https://ensaiosclinicos.gov.br/rg/RBR‐5wr2cqq

## 1 Introduction

Autism spectrum disorder (ASD) is a multifaceted neurological condition characterized by challenges in social interaction, communication, and repetitive behaviors (Hodges et al. 2020). The prevalence of ASD has been rising, leading to an urgent need for effective therapeutic interventions (Hirota and King 2023). Traditional therapies often fall short, leaving many individuals without adequate treatment options. Consequently, there is a growing interest in complementary therapies, such as the use of cannabis oil rich in cannabidiol (CBD) (da Silva Junior et al. 2022).

Social cognition refers to the neurobiological processes that enable humans and animals to interpret social cues accurately and respond appropriately (Butman and Allegri 2001). This domain encompasses a range of skills that develop progressively, including joint attention, empathy, facial expression recognition, and symbolic play (Vinic 2011). Research has consistently identified deficits in various aspects of social cognition among individuals with ASD, particularly in areas such as theory of mind and facial expression recognition (Griffin et al. 2021; Santos 2020; Shalev et al. 2022).

CBD has garnered attention for its potential therapeutic benefits, which include anti‐inflammatory, neuroprotective, and anxiolytic properties (Blessing et al. 2015; Singh et al. 2023). Emerging studies suggest that CBD may influence several neurobiological mechanisms associated with ASD, such as neurotransmitter systems, neuroinflammation, and synaptic plasticity (Walter and Stella 2004; Kwan Cheung et al. 2021). Preliminary research indicates that CBD treatment may lead to improvements in behavior, anxiety, and communication in children with ASD (Efron and Taylor 2023; Bilge and Ekici 2021; Aran et al. 2019; Barchel et al. 2019). However, comprehensive clinical trials are necessary to validate these findings and elucidate the underlying mechanisms of CBD's effects on ASD.

This manuscript presents a post hoc subanalysis of a larger randomized controlled trial previously published, focusing specifically on the effects of CBD‐rich cannabis oil on social cognitive domains in children with ASD, particularly facial expression recognition and theory of mind (da Silva Junior et al. 2024). By analyzing only the subset of children who were able to complete these neuropsychological assessments, we aim to help address the current evidence gap regarding targeted social cognition outcomes. Broader behavioral outcomes and safety findings were previously reported in the parent trial (da Silva Junior et al. 2024). The primary objective is to evaluate the effects of CBD‐rich cannabis oil on social cognitive abilities, particularly theory of mind and facial expression identification, in children with ASD. Broader behavioral and safety outcomes were previously reported in the parent trial.

## 2 Methods

### 2.1 Study Design

This is a post hoc subanalysis of a double‐blind, randomized, placebo‐controlled trial (da Silva Junior et al. 2024), conducted according to ANVISA Resolution No. 9 (Section IV, Chapter II), which regulates investigational drug trials in Brazil.

The present research received approval from the Research Ethics Committee of the Health Sciences Center at Federal University of Paraíba (UFPB), under Certificate of Presentation of Ethical Appreciation Number 89392518.4.0000.5188. The study was also registered in the Brazilian Clinical Trials Registry (ReBEC) under the ID RBR‐5wr2cqq (UTN: U1111‐1261‐4178), accessible at https://ensaiosclinicos.gov.br/rg/RBR‐5wr2cqq. All legal guardians provided informed consent, with assurance of anonymity and the right to withdraw freely.

### 2.2 Sample Size Calculation and Recruitment

Sample size was calculated using G*Power for an independent samples *t*‐test, based on an expected effect size of 0.73, *α* = 0.05, and power of 80%, using previous data from trials involving donepezil in children with ASD. Although the parent study included 62 participants, this subanalysis focused on the 19 children who completed both baseline and follow‐up social cognition assessments. No separate power analysis was conducted for this subanalysis, as it was exploratory in nature and relied on the availability of complete data within the original sample. We acknowledge that the reduced sample size limits the ability to detect small effects and interpret findings with statistical confidence. Recruitment was carried out online through autism support networks and social media platforms, with registration via a dedicated website that included completion of a sociodemographic questionnaire and the Childhood Autism Rating Scale (CARS).

### 2.3 Participants and Randomization

Eligible participants were children aged 5–11 years with a confirmed diagnosis of ASD across all severity levels (1–3). Due to cognitive limitations in the sample, child assent was not required. Exclusion criteria included epilepsy, autoimmune diseases, diabetes, systemic hypertension, and prior use of cannabis‐based products within 2 months of enrollment.

Randomization was stratified by ASD severity to ensure balanced group allocation. Alphanumeric codes were generated via random.org and managed by an external pharmacy student, the only individual aware of group assignments. This student received prior training and was supervised at all times by a licensed pharmacist who was independent of the research team. The student was responsible for labeling and delivering the study products, while all other researchers involved in the assessments remained blinded until study completion.

### 2.4 Instrumentals and Materials

As in the original trial (da Silva Junior et al. 2024), data collection began with an online questionnaire to gather sociodemographic information, later complemented by psychiatric interviews. The assessment protocol included the Childhood Autism Rating Scale (CARS) to confirm ASD diagnosis and assess symptom severity, the Social Perception/Cognition subtest of the NEPSY‐II to evaluate theory of mind, and the Facial Expression Recognition Task (FERT) to assess the recognition of emotional facial expressions. Non‐verbal intelligence was evaluated using the Raven's Colored Progressive Matrices (CPM‐Raven). Additionally, caregivers provided subjective reports regarding perceived behavioral and cognitive improvements in the children.

### 2.5 Intervention

Participants received either CBD‐rich cannabis oil (341.4 mg/g total cannabinoids; 28.9% CBD; 9:1 CBD:THC ratio; 0.5% concentration, provided by ABRACE) (CBDGp) or a placebo (PlacGp) with identical characteristics. Full details on formulation and dispensing protocols are available in the parent publication (da Silva Junior et al. 2024).

### 2.6 Clinical Protocol

Following the procedures adopted in the original trial (da Silva Junior et al. 2024), clinical procedures began with laboratory evaluations (renal, hepatic, and glycemic panels), followed by neuropsychological assessments conducted in two sessions of approximately 50 min each. A child psychiatrist led all evaluations, assisted by an assistant psychologist and a psychology student. Throughout the 12‐week treatment period, families received continuous support, including pharmacotherapeutic guidance and psychiatric follow‐up through both in‐person and telehealth consultations. This subanalysis included only the children who completed both pre‐ and post‐intervention social cognition assessments.

### 2.7 Statistical Analysis

Data was collected over 5 months. The analysis aimed to compare group means over time (pre‐ and post‐intervention). Due to violations of normality, homoscedasticity, and independence of errors in some variables, non‐parametric tests were used. The Mann–Whitney U test assessed between‐group differences, and the Wilcoxon signed‐rank test evaluated within‐group changes. Analyses were conducted using SPSS 21.0 and Excel 2013, with a 95% confidence interval.

## 3 Results

### 3.1 Sample Characterization and Sociodemographic Data

This subanalysis included only the subset of 19 children from the original 60‐participant trial (da Silva Junior et al. 2024) who were able to complete the social cognition assessments (Figure 1). As seen in Table 1, the mean age was 7.82 (± 1.99) years, with 72.7% male. Most were enrolled in school at the appropriate grade level (90.9%). Regarding ASD severity, 78.9% were classified as level 1 and 21.1% as level 2; no children with level 3 participated, as they were unable to complete the tasks. Multidisciplinary care was reported in 63.2% of cases, mostly within the public healthcare system (31.6%). The most frequent therapies were psychopedagogy (36.8%), psychology (36.8%), speech therapy (31.6%), and occupational therapy (31.6%). At baseline, 47.3% of children used psychotropic medication‐21% in the CBDGp and 26.3% in the PlacGp‐primarily risperidone (42.1%) (Table 2). Two children (one per group) discontinued medication during the study, and one participant in the PlacGP initiated psychotropic use during follow‐up. To address potential confounding, we repeated the main statistical analyses excluding these three participants. The results remained unchanged, indicating that these medication changes did not affect the overall outcomes of the study.

**FIGURE 1:**
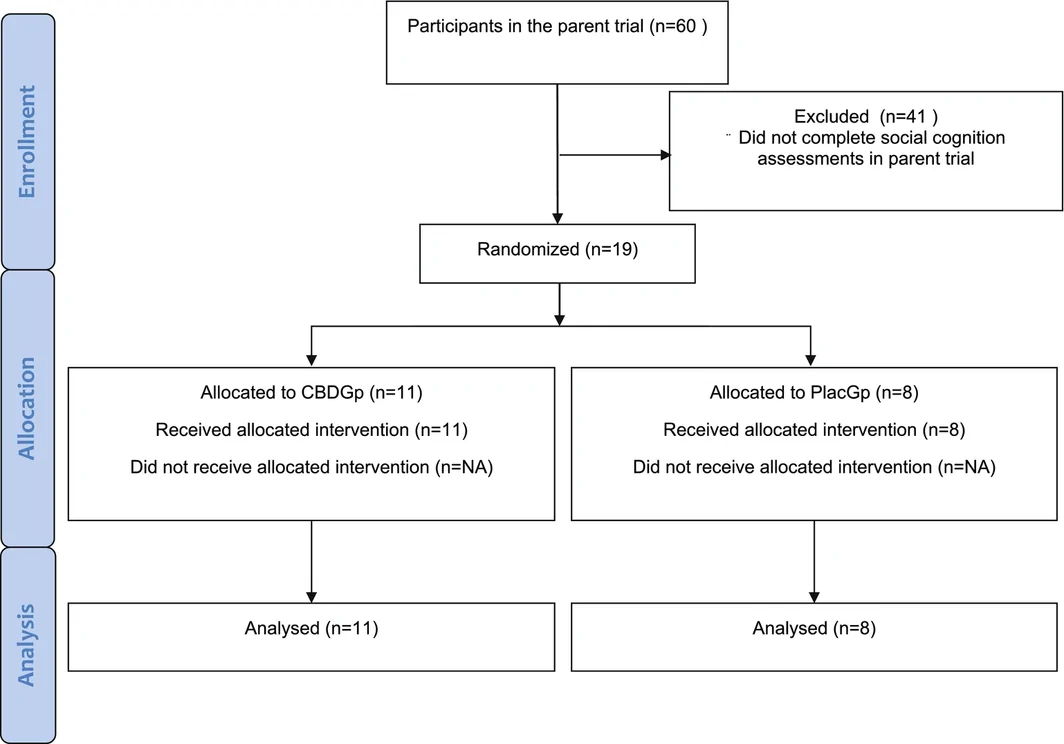
CONSORT flow diagram. CONSORT flow diagram Illustrating the enrollment, allocation, follow‐up, and analysis of participants included in this subanalysis, derived from a randomized controlled trial involving 60 participants. CBDGp, cannabidiol group; PlacGp, placebo group.

**TABLE 1:** Sociodemographic characteristics.

Measure	CBDGp (*N* = 11)	PlacGp (*N* = 8)	Chi‐squared test
Sex			
Male	8 (72.70%)	7 (87.50%)	*p* = 0.435
Female	3 (27.30%)	1 (12.50%)	
Age	7.82 ± 1.99	8.25 ± 1.66	
Children's education			
Does not attend	0 (0%)	0 (0%)	—
Below expected grade level	1 (9.10%)	1 (12.50%)	—
At expected grade level	10 (90.90%)	7 (87.50%)	—
Type of treatment			
Does not receive	3 (27.30%)	4 (50.00%)	—
Public	5 (45.50%)	1 (12.05%)	—
Private	2 (18.20%)	3 (37.50%)	—
Mixed	1 (9.10%)	0 (0.00%)	
Type of therapy			
Occupation therapy	4 (36.40%)	2 (25%)	—
Physiotherapy	0 (0.00%)	0 (0.00%)	—
Phonoaudiology	4 (36.40%)	2 (25.00%)	—
Psychology	4 (36.40%)	3 (37.59%)	—
Psychopedagogy	6 (54.50%)	1 (12.50%)	—
Others	0 (0.00%)	1 (12.50%)	—
Use of psychotropics			
No	6 (54.50%)	2 (25.00%)	—
Yes, and continues	3 (27.30%)	5 (62.50%)	—
Used and stopped	1 (9.10%)	1 (12.50%)	—
Did not use and started	1 (9.10%)	0 (0.00%)	—
ASD severity			
Level 1	8 (72.70%)	7 (87.50%)	*p* = 0.435
Level 2	3 (27.30%)	1 (12.50%)	
Level 3	0 (0.00%)	0 (0.00%)	

*Note:* Age is represented in Mean ± Standard Deviation. Other measures are represented in number of individuals (percentage of individuals). The *p*‐values of Chi‐square test are also shown for sex distribution and ASD severity.

Abbreviations: CBDGp, cannabidiol group; *N*, number of participants; and PlacGp, placebo group.

**TABLE 2:** Drugs used by children during the clinical trial.

Drug	CBD Gp (*N* = 11)	PlacGp (*N* = 8)	Total (*N* = 19)
Risperidone	4 (36.36%)	4 (50.00%)	8 (42.10%)
Methylphenidate	0 (0.00%)	1 (12.50%)	1 (5.20%)
Levomepromazine	0 (0.00%)	1 (12.50%)	1 (5.20%)
Clomipramine	0 (0.00%)	1 (12.50%)	1 (5.20%)

*Note:* Data are presented as number of participants (percentage of participants).

Abbreviations: CBDGp, cannabis group; *N*, number of participants; and PlacGp, placebo group.

### 3.2 Dosage Information, Side Effects, Subjective Improvement, and Impact of Social Isolation

An analysis of the dosages used is detailed in Table 3. Children in the CBDGp received a higher mean daily dose (47.42 drops/day ≈ 12 mg of CBD) than those in the PlacGp (40.96 drops/day ≈ 10 mg). Adverse effects were minimal and limited to two PlacGp participants (colic and acne). No adverse events were reported in the CBDGp. Subjective improvement, based on caregiver reports, was more frequently noted in the CBDGp: 63.6% of parents reported general improvement, compared to 25% in the PlacGp. In the PlacGp, 37.5% reported no change, and another 37.5% were uncertain; in the CBDGp, these rates were both 18.2%.

**TABLE 3:** Dosage information, side effects and subjective improvement.

Measures	CBDGp (*N* = 11)	PlacGp (*N* = 8)	*p*
Number of drops used	54.36 ± 10.65	45.50 ± 17.84	*p* = 0.339
Side effects	2 (18.18%)	0 (0.00%)	—
Subjective improvement			
No	2 (18.18%)	3 (37.50%)	*p* = 0.286
Uncertain	2 (18.18%)	3 (37.50%)	
Yes	7 (63.64%)	2 (25.00%)	

*Note:* Number of drops used is presented in Mean ± Standard Deviation. Side effects and subjective improvement results are presented as number of participants (percentage of participants). Subjective improvement was analyzed using the chi‐square test, and group differences in drop count were assessed with the Mann–Whitney test.

Abbreviations: CDBGp, cannabidiol group; *N*, number of participants; and PacGp, placebo group.

### 3.3 Baseline Non‐Verbal Intelligence Profile

The CPM‐Raven was applied at baseline only, due to limitations during the COVID‐19 period. Most children (36.8%) scored in the extremely low IQ range (IQ < 70). Both groups had an equal number of children with average IQ (*n* = 3), and group comparisons revealed no statistically significant differences in IQ classifications or mean percentiles (Table 4).

**TABLE 4:** IQ classification on baseline for each group.

IQ score	CBDGp (*n* = 11)	PlacGp (*n* = 8)	Total (*n* = 19)	Mann Whitney
Extremely low	4 (36.37)	3 (37.50)	7 (36.84)	—
Borderline	0 (0.00)	0 (0.00)	0 (0.00)	—
Low average	2 (18.18)	2 (25.00)	4 (21.05)	—
Average	3 (27.27)	3 (37.50)	6 (31.59)	—
High average	2 (18.18)	0 (0.00)	2 (10.52)	—
Superior	0 (0.00)	0 (0.00)	0 (0.00)	—
Percentile IQ score	34.09 ± 27.83	23.39 ± 27.30	29.58 ± 27.37	*p* = 0.229

*Note:* IQ Classification data are shown in number of participants (percentage of participants). Percentile IQ score results are shown in Mean ± Standard Deviation. The *p*‐value from Mann–Whitney test is also shown.

Abbreviations: CBDGp, cannabidiol group; PlacGp, placebo group.

### 3.4 ASD Symptom Severity

CARS scores did not differ significantly between CBDGp and PlacGp either before or after the intervention (Table 5). Likewise, no significant within‐group changes were observed in mean CARS scores from pre‐ to post‐treatment.

**TABLE 5:** Comparison of CARS between before and after clinical protocol.

Group	Before clinical protocol	After clinical protocol	Wilcoxon test	Size effect
PlacGp	36.07 ± 10.32	33.88 ± 9.14	*p* = 0.248	−0.41
CBDGp	32.20 ± 6.94	28.73 ± 7.05	*p* = 0.342	−0.29
Mann–Whitney test	*p* = 0.434	*p* = 0.214	—	—

*Note:* Results are shown in Mean ± Standardized Deviation. Size effects and *p*‐value for Wilcoxon and Mann–Whitney tests are also shown.

Abbreviations: CBDGp, cannabidiol group; PlacGp, placebo group.

### 3.5 Facial Expression Recognition and Theory of Mind Evaluation

As shown in Table 6, within‐group analyses demonstrated improvements in selected social cognition outcomes in the CBD group. At baseline, the CBDGp had a lower mean score on the Facial Expression Recognition Test (11.82 ± 3.09) than the PlacGp (13.38 ± 3.62). Following the 12‐week intervention, facial expression recognition improved significantly within the CBDGp (13.18 ± 2.48; *p* = 0.030; effect size = −0.46), whereas no significant within‐group change was observed in the PlacGp (*p* = 0.666; effect size = −0.11). However, the post‐treatment between‐group comparison was not statistically significant (*p* = 0.527).

**TABLE 6:** Facial expression recognition and theory of mind tests results comparison between groups before and after clinical protocol.

Test	Group	Before intervention	After intervention	Wilcoxon test	Size effect
Facial expression recognition test	PlacGp	13.38 ± 3.62	13.25 ± 3.91	*p* = 0.666	−0.11
	CDBGp	11.82 ± 3.09	13.18 ± 2.48	*p* = 0.030	−0.46
Mann–Whitney test		*p* = 0.168	*p* = 0.527	—	—
Verbal task of theory of mind	PlacGp	10.38 ± 5.52	12.25 ± 5.92	*p* = 0.088	−0.43
	CBDGp	9.55 ± 5.27	11.82 ± 3.97	*p* = 0.016	−0.51
Mann–Whitney test		*p* = 0.710	*p* = 0.901	—	—
Total score of theory of mind	PlacGp	14.00 ± 6.18	16.50 ± 6.82	*p* = 0.027	−0.55
	CBDGp	13.36 ± 6.43	16.09 ± 4.84	*p* = 0.020	−0.50
Mann–Whitney test		*p* = 0.620	*p* = 0.967	—	—

*Note:* Results are shown in Mean ± Standardized Deviation. Size effects and *p*‐value for Wilcoxon and Mann–Whitney tests are also shown.

Abbreviations: CBDGp, cannabidiol group; PlacGp, placebo group.

A similar pattern was observed for verbal theory of mind. Scores improved significantly from baseline (9.55 ± 5.27) to post‐intervention (11.82 ± 3.97; *p* = 0.016; effect size = −0.51) within the CBDGp, whereas the within‐group change in the PlacGp did not reach statistical significance (*p* = 0.088; effect size = −0.43). Nevertheless, there was no statistically significant difference between groups after the intervention (*p* = 0.901).

For total theory of mind scores, significant within‐group improvements were observed in both the PlacGp (*p* = 0.027; effect size = −0.55) and CBDGp (*p* = 0.020; effect size = −0.50). The between‐group difference remained non‐significant (*p* = 0.967).

## 4 Discussion

The findings provide a nuanced picture of changes in social cognition following CBD‐rich cannabis oil treatment in children with ASD. Although significant within‐group improvements were observed in facial expression recognition and verbal theory of mind in the CBDGp, these changes were not accompanied by statistically significant differences compared with the placebo group. Similarly, total theory of mind scores improved significantly within both groups, with no evidence of a between‐group difference. Therefore, the present findings should not be interpreted as demonstrating superiority of CBD‐rich cannabis oil over placebo. Rather, they identify preliminary within‐group changes that warrant further investigation in adequately powered trials.

The lack of significant differences in the broader ASD symptomatology, as measured by CARS, is worth noting. These scales encompass a wide range of behaviors and skills, and the absence of broad changes indicates that CBD may not uniformly affect all ASD‐related symptoms. Instead, its impact might be more targeted, enhancing specific cognitive functions rather than causing widespread behavioral changes (Fusar‐Poli et al. 2020; Aran et al. 2021). This finding emphasizes the need for more targeted measures and assessments in future studies to fully capture the effects of CBD on the diverse manifestations of ASD.

The improvements observed in facial expression recognition and theory of mind in the CBDGp are particularly intriguing, given the central role these cognitive abilities play in social communication (Ruihua et al. 2021). Facial expression recognition is critical for interpreting social cues, while theory of mind involves understanding the mental states of others, both of which are often impaired in individuals with ASD (Altschuler et al. 2021; Loth et al. 2018). Although the within‐group improvements observed in the CBDGp raise hypotheses regarding possible effects on neural processes involved in social cognition, the absence of significant between‐group differences prevents attribution of these changes specifically to CBD. Nevertheless, potential mechanisms may be considered in light of previous experimental evidence, including modulation of the endocannabinoid system and its involvement in mood, memory, emotional processing, and social behavior (Tudorancea et al. 2022; Wei et al. 2017).

The underlying mechanisms by which CBD might exert these effects remain an area of active investigation. Preclinical studies suggest that CBD's interaction with the endocannabinoid system could influence synaptic plasticity, neurotransmitter release, and neuroinflammation–factors that are thought to contribute to the pathophysiology of ASD (Galaj and Xi 2020; Martinez Naya et al. 2023; Ibeas Bih et al. 2015). Additionally, CBD's anxiolytic and neuroprotective properties may reduce social anxiety and enhance cognitive flexibility, further supporting its potential role in improving social cognition in children with ASD (Schouten et al. 2024; Korb et al. 2023).

Cannabinoid receptors play a role in emotional processing and are located in limbic regions, including the amygdala, the anterior cingulate cortex, the hippocampus, and the frontal cortex (Hindocha et al. 2015). In a fMRI study involving healthy adults who were administered isolated CBD, isolated THC, or a placebo, it was observed that CBD—unlike THC—reduced connectivity between the anterior cingulate cortex and the amygdala during emotional processing of fear‐related stimuli. These findings support the neurophysiological correlates of CBD's anxiolytic properties and also highlight additional potential benefits of the substance in relation to emotions or the recognition of facial expressions (Fusar‐Poli et al. 2010).

Research has also been conducted to explore how the therapeutic effects of anxiolytics and antidepressants may enhance facial expression recognition. Given that phytocannabinoids exhibit anxiolytic activity, a systematic review assessed the effects of CBD and THC on facial expression recognition (Rossi et al. 2020). The authors identified 10 studies but noted significant heterogeneity among them regarding administration, product type, dosage, and delivery method. The results indicated that seven of the 10 studies reviewed found no significant effect of phytocannabinoids on facial expression recognition. However, all studies were conducted with healthy individuals, and no data were available on facial expression recognition among individuals with autism following the use of medicinal cannabis.

Improvements in facial expression recognition, with an effect size of 60%, were noted in adult users after CBD use. In contrast, the isolated use of THC significantly impaired recognition of faces, with an effect size of 40%. The combination of CBD and THC appeared to mitigate the impairing effects of THC. The authors suggested that cannabinoids influence emotional processing, with CBD subtly enhancing facial emotional recognition and protecting against deficits caused by THC (Hindocha et al. 2015).

Regarding theory of mind, a study found that participants under the influence of CBD performed better on cognitive theory of mind tasks compared to those who received a placebo, although this advantage was not observed in comparisons with THC or THC + CBD, providing evidence for the interaction between the effects of THC and CBD on social cognitive abilities (Sainz‐Cort et al. 2024). However, the absence of significant changes in global ASD symptoms indicates that these effects might be domain‐specific, reinforcing the complexity of ASD and the need for a multifaceted approach to treatment.

The administration of CBD‐rich cannabis oil was generally well‐tolerated by the participants, with minimal side effects reported. The higher daily drop count in the CBDGp suggests that the dosage was manageable for caregivers and did not lead to significant adherence issues (Vidot et al. 2017).

The study found some limitations, such as a small sample size, inclusion of children with varying ASD severity, and the reliance on parent‐reported outcomes. Additionally, the lack of a follow‐up period hinders evaluation of long‐term cognitive improvements.

## 5 Conclusion

This exploratory post hoc subanalysis identified significant within‐group improvements in facial expression recognition and verbal theory of mind among children receiving CBD‐rich cannabis oil. However, these changes were not significantly different from those observed in the placebo group, and total theory of mind scores improved within both groups. Accordingly, the findings do not establish a specific treatment effect of CBD‐rich cannabis oil on social cognition in children with ASD. Given the small sample size and exploratory design, larger adequately powered randomized trials with longer follow‐up are needed to determine whether CBD‐rich cannabis oil provides clinically meaningful benefits beyond placebo.

## Funding

This work was supported by Coordination for the Improvement of Higher Education Personnel (CAPES).

## Ethics Statement

The present research received approval from the Research Ethics Committee of the Health Sciences Center at Federal University of Paraíba, under Certificate of Presentation of Ethical Appreciation Number 89392518.4.0000.5188. The study was also prospectively registered in the Brazilian Clinical Trials Registry (ReBEC) under ID RBR‐5wr2cqq (UTN: U1111‐1261‐4178), accessible at https://ensaiosclinicos.gov.br/rg/RBR‐5wr2cqq.

## Conflicts of Interest

The authors declare no conflicts of interest.

## Data Availability

The data that support the findings of this study are available on request from the corresponding author. The data are not publicly available due to privacy or ethical restrictions.
